# Academy of Stomatology—Resolutions of Respect to Henry H. Burchard, M.D., D.D.S.

**Published:** 1901-02

**Authors:** 


### ACADEMY OF STOMATOLOGY—RESOLUTIONS OF RESPECT TO HENRY H. BURCHARD, M.D., D.D.S.

At the regular meeting of the Academy of Stomatology, held Tuesday evening, October 23, 1900, the Committee on Resolutions upon the death of Dr. Henry H. Burchard submitted the following, which were accepted and adopted:

Whereas, Henry H. Burchard, M.D., D.D.S., has been removed by death from the scene of his toils and his honors; therefore be it

*Resolved*, As the sense of this society, that in the death of Dr. Burchard the Academy of Stomatology has lost one of its most brilliant and useful members and the dental profession one of its most earnest workers.

As one of its organizers he was foremost in the endeavor to establish the Academy of Stomatology upon a plane of high professional usefulness, and was ever active in advancing its interest by contributing his own work and enlisting the co-operation of others. He gave unselfishly of his energies and best endeavors even when physically unfitted for the task. His active brain not only stimulated thought and discussion at the sessions of the Academy, but his suggestive help in the ordering of its affairs was always a material aid in its progress.

He was earnest and enthusiastic in his efforts to impart his knowledge to others. As a teacher he was clear, logical, and forcible. These qualities he evinced both as a writer and as a speaker.

As a man Dr. Burchard was genial and affable in disposition, ever ready to sacrifice his own time and strength whenever it was within his power to aid others, and especially those who were earnestly working in the field of dental advancement. In his brief and brilliant career as writer and teacher he had attained remarkable distinction, and though his untimely decease has occurred at an age when the promise of still greater achievements seemed clearly before him, he nevertheless attained an eminence in his profession as teacher, writer, and investigator seldom reached by others, and less frequently by those of his short period of life.

*Resolved,* That these resolutions be spread upon the minutes of the Academy, and that a copy be transmitted to his family and be published in the dental journals.

EDWIN T. DARBY,
S. H. GUILFORD,
EDWARD C. KIRK,
*Committee.*
ARCHIBALD C. EGLIN,
*Secretary.*

